# Smaller species experience mild adversity under shading in an old‐field plant community

**DOI:** 10.1002/ece3.9006

**Published:** 2022-06-22

**Authors:** Kelly C. Balfour, Danielle A. Greco, Riley Gridzak, Gillian Piggott, Brandon S. Schamp, Lonnie W. Aarssen

**Affiliations:** ^1^ Department of Biology Queen’s University Kingston Ontario Canada; ^2^ 7236 Department of Biology Algoma University Sault Ste. Marie Ontario Canada

**Keywords:** determinants of plant community diversity and structure, herbaceous canopy, light competition, photosynthetically active radiation, shade, size advantage

## Abstract

Plant competition experiments commonly suggest that larger species have an advantage, primarily in terms of light acquisition. However, within crowded natural vegetation, where competition evidently impacts fitness, most resident species are relatively small. It remains unclear, therefore, whether the size advantage observed in controlled experiments is normally realized in habitats where competition is most intense. We characterized the light environment and tested for evidence of a size advantage in competition for light in an old‐field plant community composed of perennial herbaceous species. We investigated whether larger species contributed to reduced light penetration (i.e., greater shading), and examined the impact of shade on smaller species by testing whether their abundance and richness were lower in plots with less light penetration. Light penetration in plots ranged from 0.3% to 72.4%. Significant effects were more common when analyses focused on small plants that reached reproduction (i.e., flowering rooted units); focusing on only flowering plants (i.e., excluding nonflowering rooted units) can clarify community patterns. Plots with a greater mean species height had significantly lower light penetration, and plots with lower light penetration had significantly lower flowering abundance and richness of small species. However, the impact of shade on the flowering abundance and richness of small species was relatively small (*R* ^2^ values between 8% and 15%) and depended on how we defined “small species.” *Synthesis*: Our results confirm that light penetration in herbaceous vegetation can be comparable to levels seen in forests, that plots with taller species cast more shade, and that flowering smaller species are less abundant and diverse in plots where light penetration is low. However, variation in mean plot height explained less than 10% of variation in light penetration, and light penetration explained between 5 and 15% of variation in the flowering abundance and richness of small species. Coupled with the fact that flowering small species were present even within the most heavily shaded plots, our results suggest that any advantage in light competition by large species is limited. One explanation is that at least some small species in these communities are shade‐tolerant. Shade tolerance in predominantly herbaceous communities, particularly among small plant species, requires further research.

## Graphical abstract

We characterized the light environment and tested for evidence of a size‐advantage in light competition in an old‐field plant community. We found that plots with taller species cast more shade, and that smaller species were less abundant and diverse in plots where light penetration was low; however, variation in mean plot height explained less than 10% of variation in light penetration, light penetration explained 5‐15% of variation in small species abundance and richness, and flowering small species were present even within the most heavily shaded plots. Our results suggest that any advantage in light competition by large species is limited.

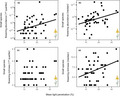

## 1 INTRODUCTION

Evidence from neighbor removal studies clearly indicates that plant communities are highly competitive (e.g., Clements et al., 1929; Grime, 1973; Gurevitch et al., 1992; Tilman, 1989). Research also indicates that variation in plant functional traits is important in determining competitive ability and predicting the suppression of neighbor growth (Aarssen & Keogh, 2002). For example, larger plant species are generally considered superior to smaller species when competing for pollinators (e.g., Donnelly et al., 1998), seed dispersal agents (e.g., Thomson et al., 2011), and for light (e.g., Schwinning & Weiner, 1998; Weiner, 1990). We refer to the competitive advantage commonly attributed to relatively large/tall plant species as the “size advantage hypothesis” (e.g., Tracey & Aarssen, 2014; Tracey et al., 2017).

Light is an essential resource, and light limitation can negatively impact plant growth (Bazzaz, 1979; Grubb, 1998; Tilman, 1985). Light availability varies across the growing season (Bachmann et al., 2018), with canopy maturation (Ballaré, 1994) and with community density (Givnish, 1982). When competing species vary in size, competition for light is said to be size‐asymmetric (DeMalach et al., 2017; Lamb et al., 2009), with larger plants expected to dominate light competition by intercepting more light per unit size than smaller plants (Anten & Hirose, 1998; DeMalach & Kadmon, 2017). This growth of larger species imparts additional light limitation on smaller species while also reducing their own likelihood of being shaded (Anten, 2005). When soil resources are adequate, natural communities can support the growth of tall plant species, which are able to limit the light reaching smaller plant species. For example, soil fertilization can result in increased light limitation and reduced grassland diversity (Borer et al., 2014). However, despite a perceived size advantage in terms of expected negative impact on small species survival, growth, and reproduction, small plants remain both ubiquitous and abundant within herbaceous vegetation (Aarssen et al., 2006). There is also little evidence of deterministic organization of species according to size in herbaceous communities (Schamp et al., 2008). Recent research has shown that species with larger body size in fact do not dominate neighborhood biomass production in old‐field vegetation (Tracey et al., 2017), nor do they recruit more offspring from the soil seed bank (Tracey & Aarssen, 2019).

Light limitation has been studied extensively in forests (Canham et al., 1990, 1994; Gilliam & Roberts, 2003; Gommers et al., 2013), but whether the impact of light limitation in forests is comparable to that in herbaceous vegetation remains unclear. While grasslands and old‐fields are largely dominated by herbaceous species, distinct canopy layers have attributed vertical complexity to forests (Miedema et al., 2019; Parker & Brown, 2000). In contrast, researchers have implied higher abundance and even distribution of light within herbaceous communities. However, several studies counter this widespread assumption and collectively argue that light heterogeneity exists in herbaceous communities (Ballaré, 1994; Bazzaz, 1990; Heger, 2016; Huber et al., 2021; Kelly & Canham, 1992; Körner, 1995; Liira et al., 2002). Herbaceous vegetation exhibits a layered canopy structure (Körner, 1995; Liira et al., 2002) which, according to experimental studies, can reduce light penetration to the ground level to as low as 3% (Spehn et al., 2000), comparable to mean light levels of 4.1%–4.6% observed at the ground level of some temperate forests (Bartemucci et al., 2006). However, we struggled to find many published assessments of light penetration in herbaceous communities. One study found a reduction in diversity of small plant species in experimental plots under fertilization as ground‐level light penetration decreased (Borer et al., 2014). Other experimental studies have also assessed the impact of light availability on herbaceous vegetation (e.g., Bachmann et al., 2018; Semchenko et al., 2012); however, analyses have yet to examine light conditions in unmanipulated communities (i.e., absent of seeding, fertilization, and watering treatments).

It remains unclear how small species persist in herbaceous vegetation despite their apparent disadvantage in light competition, but the ubiquity of small species, on every scale from regional floras to local neighborhoods, suggests that these species either avoid or tolerate low‐light conditions. In woodlands, species that remain shaded under various canopy layers may minimize light requirements through high photosynthetic efficiency and low light compensation points (Ballaré et al., 1997; Boardman, 1977; Givnish, 1988; Valladares & Niinemets, 2008). In this case, there may be little to no change in small species abundance or richness associated with the amount of shade cast by taller vegetation. Smaller species may also rely on light from canopy gaps and sunflecks, which are commonly observed in forests (Chazdon & Pearcy, 1991) and are likely important within herbaceous communities as well. Greater heterogeneity in canopy height in herbaceous communities and higher frequency of disturbance can increase the incidence of canopy gaps and sunflecks, allowing more light to penetrate toward the soil surface, which may thus support a greater number of small species (Chesson and Huntley 1997; Roxburgh et al., 2004). Additionally, small species may rely on early season light availability prior to canopy closure; although seasonal variation in light has been understudied in herbaceous communities, small species have been found to flower earlier in the season (Du & Qi, 2010; Segrestin et al., 2020; Sun & Frelich, 2011). Early growth and flowering by small species may reduce competition for light, as well as for pollinators, allowing these species to coexist with larger, later flowering species (Jensen et al., 2019).

In this study, we sought to answer two important questions concerning the presumed advantage that larger species have in light competition within a temperate mesic old‐field community. First, we examined patterns of light penetration through the standing vegetation to determine whether sample plots containing taller resident species have significantly reduced light penetration. Second, we tested whether the flowering abundance and richness of smaller plant species were lower in sample plots where light penetration was lowest. The goal of this research was to better reconcile experimental evidence that large/tall plant species generally enjoy a competitive advantage for light with the widespread prevalence of small species, routinely growing with larger ones, in old‐field vegetation.

## 2 METHODS

### 2.1 Study site

We conducted this experiment from June to August 2006 within a 67.5 × 50 m old‐field (known locally as the “Cemetery Field”) located at the Queen's University Biological Station, north of Kingston, Ontario, Canada (44° 31′ 17.9″ N, 76° 23′ 07.9″ W), and consisting primarily of perennial herbaceous species. For about 50 years preceding this work, this field was harvested for hay annually (excluding 2005 and 2006) but has not otherwise been disturbed. In July 2005, the field was divided into 486 plots, each 1 × 1 m, separated by 1 m laneways to minimize the impact of edge effects and disturbance during data collection (Piggott, 2007). Of these 486 pre‐existing plots, 49 were randomly chosen for data collection in this study.

### 2.2 Data collection

Monthly, from June to August 2006, we measured light intensity (photosynthetically active radiation; PAR) within each of the 49 plots using a LI‐COR L1–250 light meter (µmol s^−1^ n^−2^; LI‐COR Biosciences). We focused on this part of the growing season to test established predictions that small plants suffer under shade. In May, the canopy remains underdeveloped and is less size‐asymmetric, and by the end of the growing season, canopy shade was reduced again, undergoing a decline in August. Furthermore, all small species in our study community had completed reproduction prior to September. Consequently, we did not measure light in May or September when shade was lowest within our community.

**FIGURE 1:**
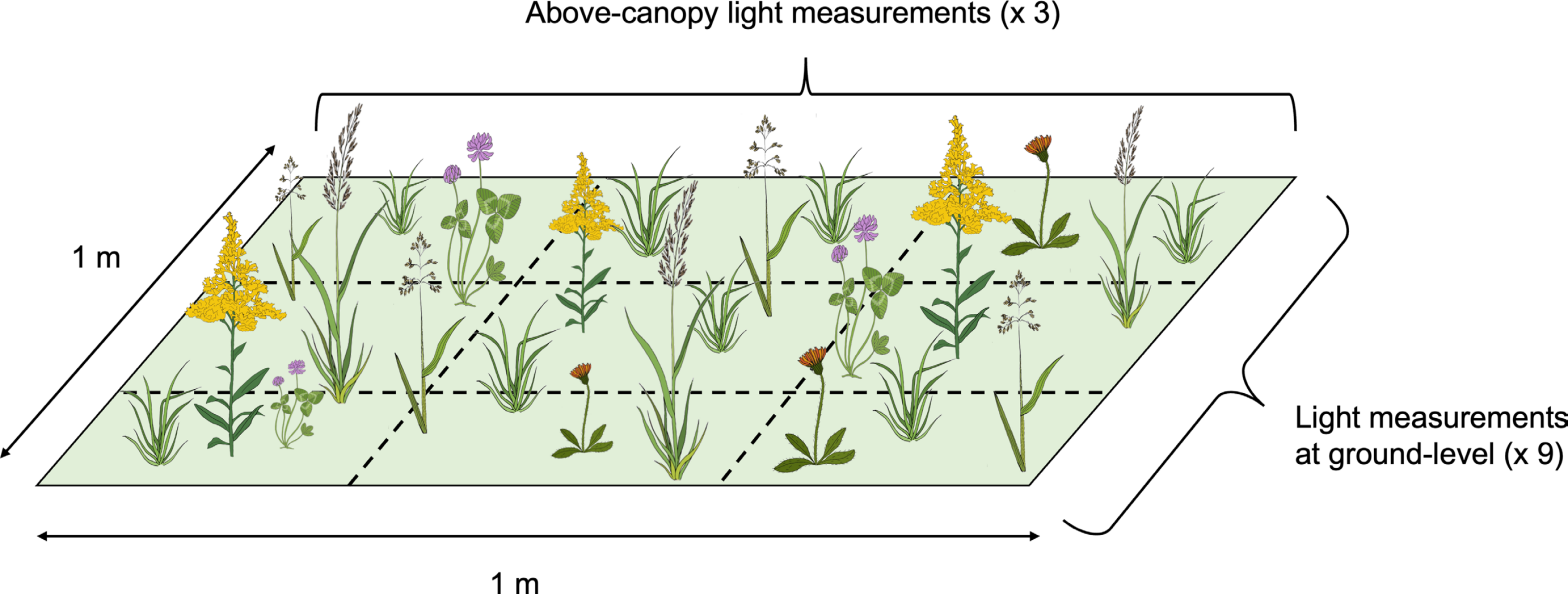
Experimental plot setup for measuring light penetration. Plots were laid out in 1 × 1 m squares divided into a 3 × 3 grid for a total of nine equal subsections within each plot. We took three light intensity measures (µmol s^−1^ n^−2^) at the center of each row directly above the canopy, with an additional nine measurements (one in each subsection) at the ground level. We then calculated three measures of percentage of light penetration for each plot by taking the mean light intensity at the ground level for each row and using the equation: 100× (ground‐level light intensity / above‐canopy light intensity)

Each plot was divided into nine equal squares in a 3 × 3 grid, with three measurements taken in the center of each plot row, directly above the canopy, and nine measurements taken at ground level in each square (Figure 1). We calculated the percentage of light penetration based on the mean light intensity at ground level for each of the nine locations in the plot and the mean above‐canopy light intensity measured above each plot row [100 × (ground‐level light intensity/above‐canopy light intensity)].

From June to August 2006, we collected abundance data by quantifying the number of rooted units per species in each plot (i.e., ramets, Schamp et al., 2016) and determined plot‐level species richness from these abundance data. As we only collected data in one year, our abundance data offers only a snapshot of species occurrence in our old‐field community, limiting our ability to fully explore species coexistence. However, our main focus was on the ability of smaller species to persist and reproduce under different light conditions, which we believe adequately reflects species responses to light conditions. We monitored plot‐level abundance biweekly, which was further classified into two census types: “total abundance” of each species (including all rooted units in a plot, both flowering and nonflowering) and “flowering abundance” of each species (including flowering rooted units in a plot). Measuring the flowering abundance for each species in each plot allowed us to better assess the ability of species to succeed (i.e., reach reproduction) under the light conditions measured for each plot. Maximum height (cm) data were collected for all species in the dataset from specimens measured at the study site (but not in our sample plots) and in nearby old‐fields at the Queen's University Biological Station, so as not to disturb the canopies of our sample plots. The number of values recorded per species ranged between 5 and 20, and the largest value per species was used as the maximum height for that species.

### 2.3 Data analyses: variation in the light environment

We quantified light penetration in our grassland community to determine how much light variation exists and to identify sources of that variation. We examined light penetration across plots to assess variation across the site (“interplot light variation”), within plots to quantify heterogeneity in light penetration (“intraplot light variation”), and across months to determine how light penetration changed as the canopy developed (“temporal light variation”). To quantify interplot light variation, we calculated mean light penetration (%) within a plot during one sampling event, and further calculated variance (σ^2^) between these means for each month (*N* = 3). For intraplot light variation, we calculated the variance in light penetration within each plot for each month (*N* = 147). Finally, to quantify temporal variation, we calculated variance in mean light penetration for each plot across months (June, July, and August; *N* = 49).

Additionally, to assess how light penetration levels differ across June, July, and August, we performed a one‐way repeated measures ANOVA using mean light penetration as the response variable and plot as the within‐subject factor, followed by a Bonferroni post hoc test to determine significant differences between months. We visually assessed normality and checked for equal variances among months using Mauchly's test for sphericity. For these analyses, we used the “anova_test” and “pairwise_t_test” functions from the package “rstatix” v0.6.0 (Kassambara, 2020).

### 2.4 Data analyses: species size effects on—and responses to—light penetration

Because there is no standard definition of “small species,” we compared two approaches: small species were regarded as those smaller than (1) the first quartile of species height for all species in the focal community (“1st quartile,” <53.25 cm, *N* = 15), or (2) the median of species height in the community (“median,” <83 cm, *N* = 29; Figure S1). Comparing results across these two definitions allowed us to test whether any significant patterns were sensitive to our method of defining small species. In addition to size, other characteristics like growth form could influence species responses to light availability. However, as only three graminoid species were included under both definitions of “small species,” and the remaining species were all forbs, we could not confidently consider these classifications in our analyses, leaving the exploration of this possibility to future work.

We tested whether plots with larger species had lower light penetration. Specifically, we fit linear models with mean plot height (mean plot‐level maximum height weighted by species plot‐level total abundance) and large species total abundance (including all species which are not considered “small” by either definition, >83 cm) as predictors and mean light penetration as the response variable. We also tested whether both the total and flowering abundances and richness of small species responded to variation among plots in light penetration. We fit linear models with the total and flowering abundances and richness of small species (for both “1st quartile” and “median” defined small species) as response variables, and light penetration and mean intraplot variation in light penetration (calculated for each plot at each sampling event and averaged across months) as our predictor variables (Table S1). We checked the variance inflation factor to ensure that there was not a high degree of multicollinearity between our variables.

We used the package “stats” from R.4.0.3 (R Core Team, 2020) for modeling, and checked statistical assumptions using residual versus fitted, normal quantile‐quantile, scale location, and constant leverage plots (“ggfortify” v.0.4.11; Tang et al., 2016). We log_10_‐transformed variables where necessary to meet assumptions (Table S1). For our multiple linear regressions, we used the Akaike Information Criterion corrected for small sample sizes for model selection (AICc). Where multiple models were within two units of the lowest AICc score, we used the full model average (via the “model.avg” function from the “MuMIn” v1.43.17 package; Bartoń, 2020) to determine the significance of predictors.

Finally, to understand the collective response of small species to light availability, we performed distance‐based redundancy analyses (dbRDA) using Bray–Curtis dissimilarities calculated from abundances of each small species per plot. We used the “decorana” function from the package “vegan” v.2.5‐6 (Oksanen et al., 2019) to confirm linearity of responses. We conducted dbRDA with mean light penetration and mean intraplot variation as possible predictors using the “capscale” function from “vegan” v.2.5‐6 (Oksanen et al., 2019) to assess how the flowering abundance of each small species changed in ordinal space along fixed axes of mean light penetration and mean intraplot variation. This allowed us to ascertain how much variation in the flowering abundances of each small species across the community was driven by light availability. We compared models using AICc scores, selecting the model with the lowest score.

## 3 RESULTS

We performed analyses using both total and flowering abundance; however, because the analyses which used only flowering abundance data were more informative, we have focused on these (i.e., we have excluded nonflowering rooted units from the results reported below). However, measures of plot‐level mean height were determined from both flowering and nonflowering plant data, as both impact light penetration. We provide further information on analyses completed using the total number of individuals (i.e., both flowering and nonflowering rooted units) in Figures S5–S8.

### 3.1 Variation in the light environment

**FIGURE 2:**
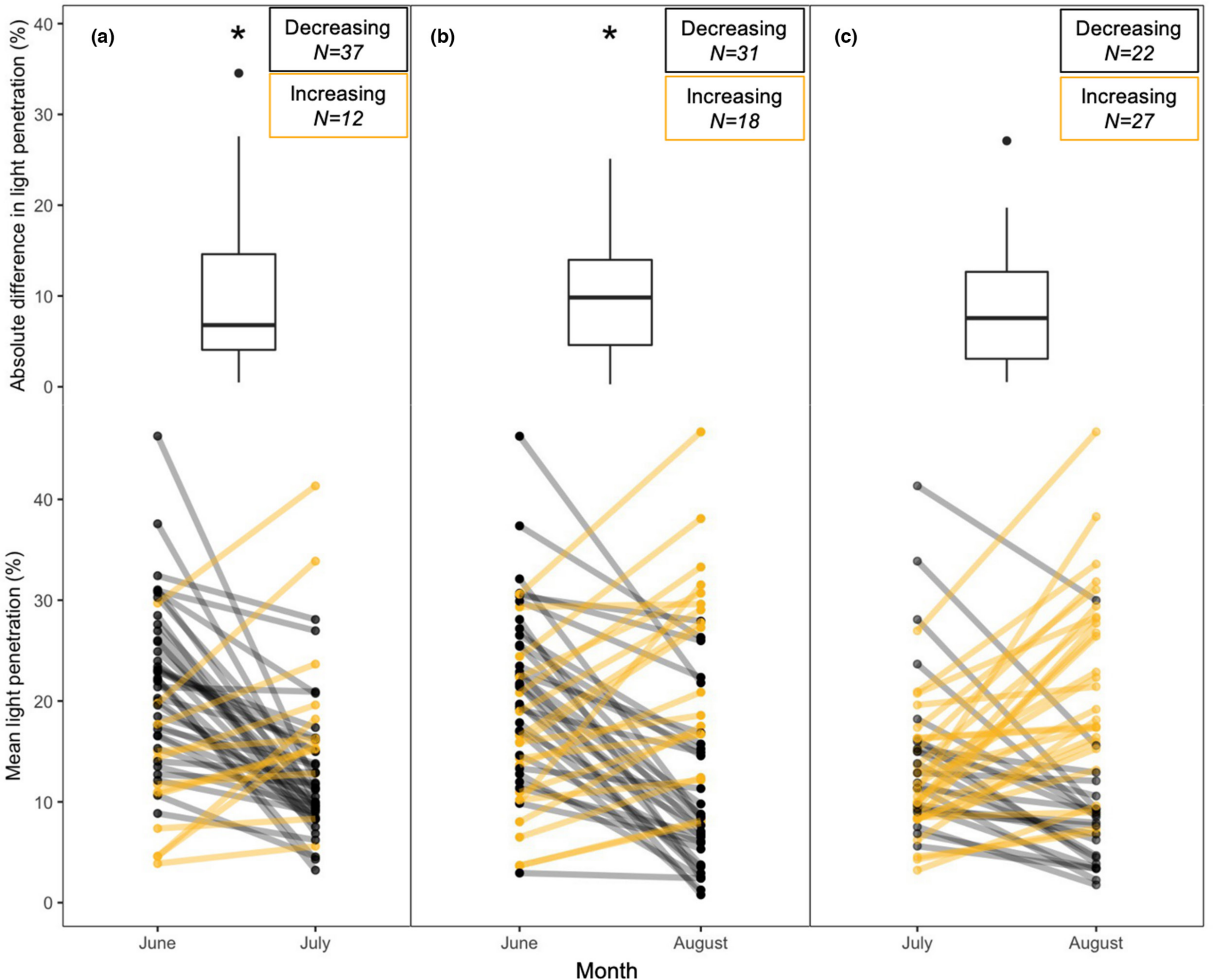
Comparisons of light penetration (%) between June and July (a), June and August (b), and July and August (c). Boxplots show absolute differences in light penetration within each plot between month pairings. Line plots show a change in mean light penetration for each plot between month pairings. Mean light penetration at the plot level was significantly different between months (repeated measures ANOVA, *F*_2,96_ = 9.2, *p* < .001). Mean light penetration in June was significantly higher than in July (Bonferroni, *P*_adj_ < .001) and August (Bonferroni, *P*_adj_ = .036). July and August did not significantly differ (Bonferroni, *P*_adj_ = .4). Between June and July, 37 plots decreased in light penetration while 12 plots increased. Between June and August, 31 plots decreased in light penetration while 18 plots increased. Between July and August, 22 plots decreased in light penetration while 27 plots increased. Boxes represent the 25th to 75th percentiles of the data, and whiskers represent the 10th and 90th percentiles. Lines colored in black represent plots that decreased in light penetration from the first to the second month, and lines colored in yellow represent plots that increased in light penetration from the first to the second month. Month pairings marked by an asterisk (*) significantly differed in mean light penetration

Light penetration within our old‐field site ranged from 0.3 to 72.4% (*n* = 440, *μ* = 16.6% ±12; Figure S2A). The highest variance in light penetration occurred between plots (*n* = 3, median interplot variance = 77.3) followed by variance across months (*n* = 49, median temporal variance = 57.4), with within‐plot light penetration varying the least (*n* = 147, median intraplot variance = 26.2; Figure S3). Intraplot variance in light penetration ranged from 0.03 to 1546.5 (*n* = 147, *μ* = 82.8 ± 17.5; Figure S2B). Mean light penetration was highest in June (20.2%) and lowest in July (13.6%), but slightly higher in August (15.9%). Light penetration differed significantly across months (repeated measures ANOVA, *F*_2,96_ = 9.2, *p* < .001; Figure 2), with a post hoc test revealing that light penetration in June was significantly different than in both July (Bonferroni, *P*_adj_ < .001; Figure 2a) and August (Bonferroni, *P*_adj_ = .036; Figure 2b), but that July and August did not significantly differ (Bonferroni, *P*_adj_ = .4; Figure 2c). High variance in light penetration among sample plots further justified our plot‐level approach to hypothesis testing.

### 3.2 Species size and light penetration

**FIGURE 3:**
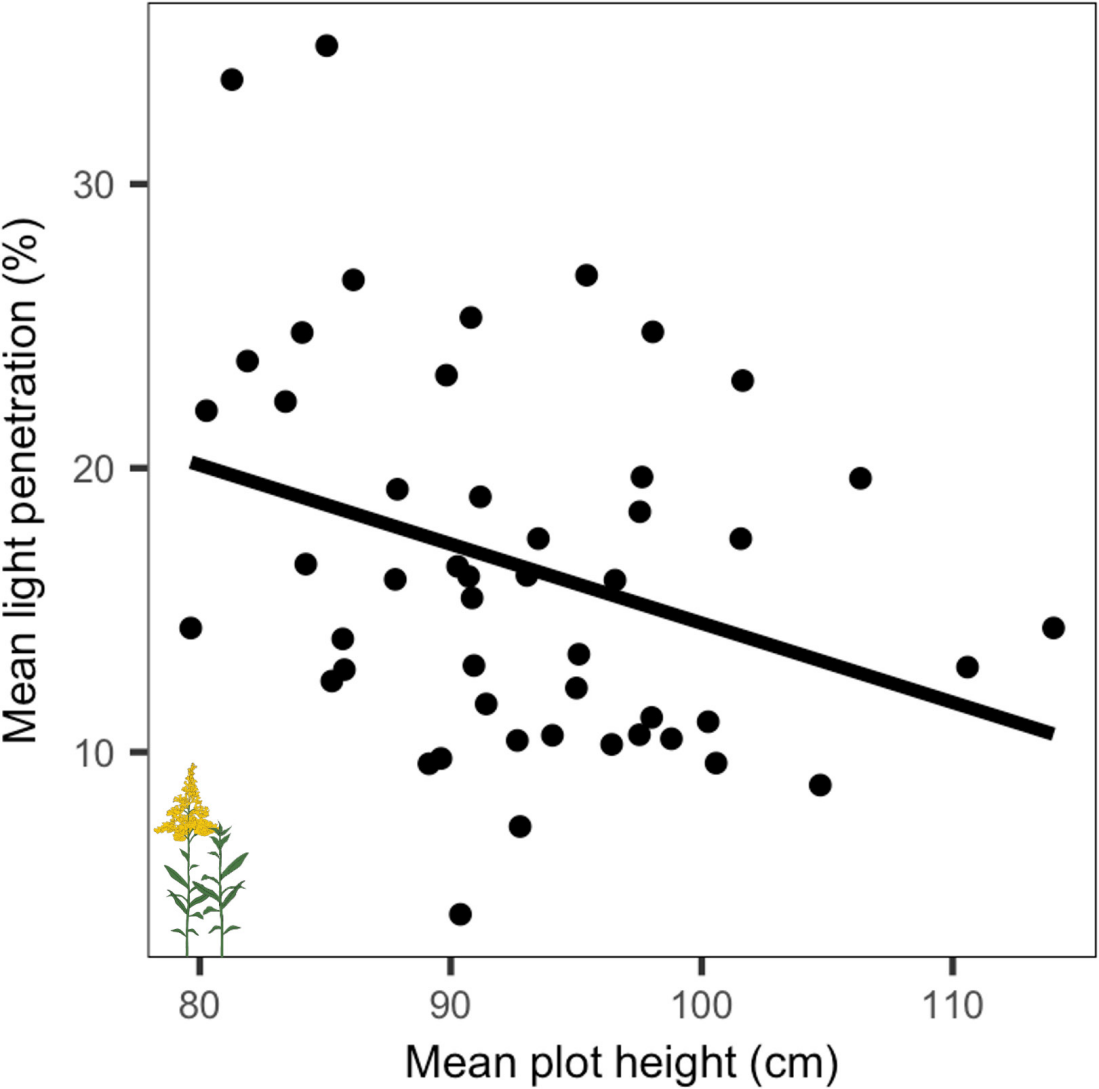
Mean plot‐level maximum height weighted by species plot‐level total abundance (as indicated by the flowering and nonflowering icons; “mean plot height,” cm) versus mean light penetration (%) per plot. We included both flowering and nonflowering individuals, as both reduce light penetration to the ground level via light interception. Mean light penetration significantly declined with mean plot height (*p* = .025, *R* ^2^ = .08)

Light penetration was significantly lower in plots with greater plot‐level mean height (*R* ^2^ = .08, *p* = .025; Figure 3) but was unassociated with the total abundance of large species. Mean intraplot variation in light penetration was significantly positively correlated with mean light penetration (Spearman correlation, *rho* = 0.49, *p* < 0.001; Figure S4).

**FIGURE 4:**
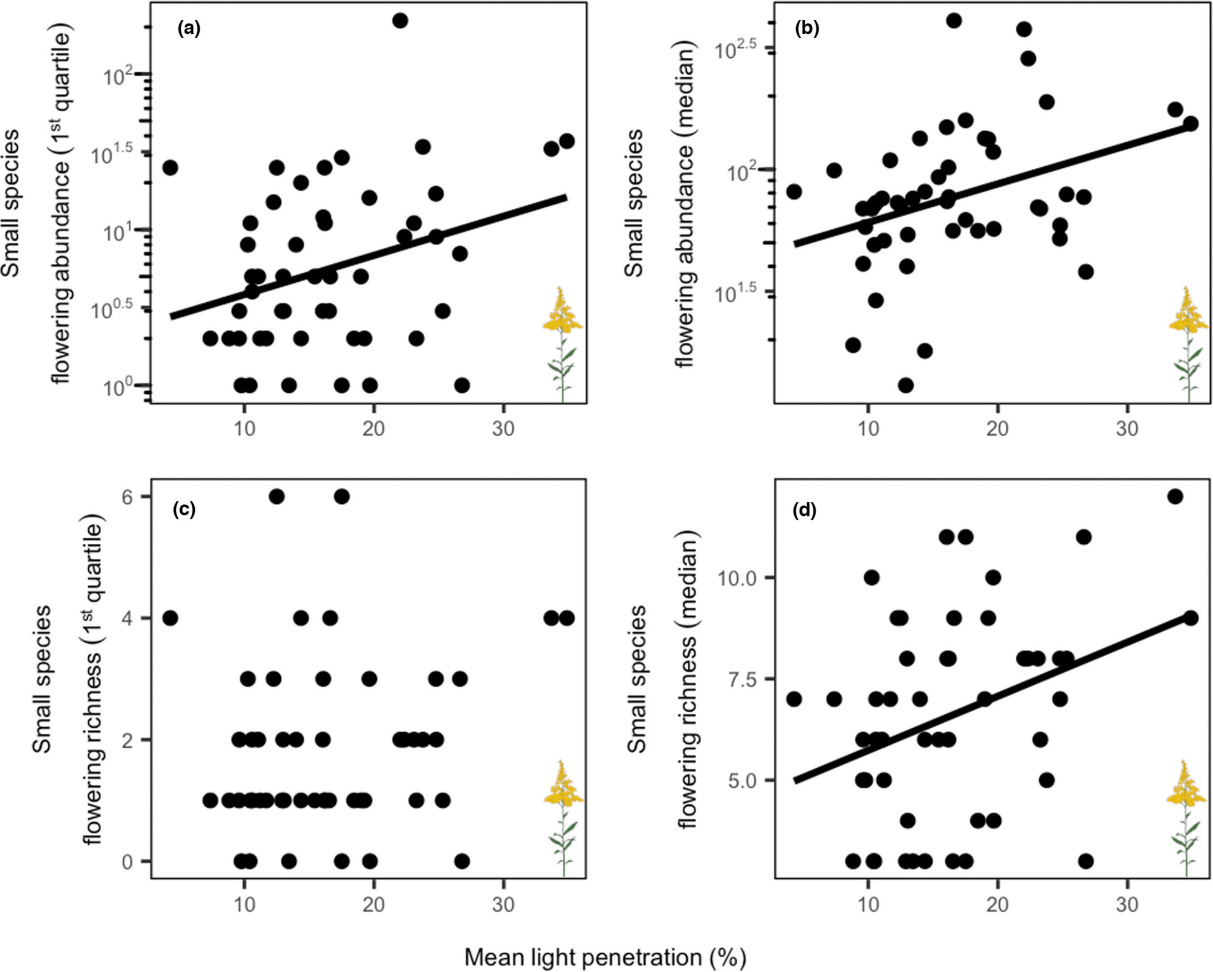
Flowering (as indicated by the flowering icon) abundance of small species under the first quartile height (“1st quartile,” a), flowering abundance of small species under the median height (“median,” b), flowering richness of “1st quartile” small species (c), and flowering richness of “median” small species (d) versus mean light penetration (%) per plot. We used log_10_ transformations on the flowering abundances of “1st quartile” and “median” small species and fit all with linear models. Flowering abundance of “1st quartile” small species (A; *p* = .027, *R* ^2^ = .08), flowering abundance of “median” small species (B; *p* = .012, *R* ^2^ = .11) as well as flowering richness of “median” small species (D; *p* = .014, *R* ^2^ = .10) significantly declined as mean light penetration decreased. Flowering richness of “1st quartile” species was not significantly affected by light penetration (c)

The abundance and richness of small species that flowered in our study year (i.e., flowering abundance and richness) were significantly higher in plots with high light penetration, but not for both definitions of small species. Small species under the “1st quartile” definition had significantly higher flowering abundance (i.e., there were more flowering small plants) in plots with higher light penetration (*R* ^2^ = .08, *p* = .027; Figure 4a), but “1st quartile” flowering richness did not significantly change with light penetration (Figure 4c, Table S1). “Median” flowering abundance and richness were both significantly higher in plots with higher light penetration (Abundance: *R* ^2^ = .1, *p* = .012; Richness: *p* = .014, *R* ^2^ = .1; Figure 4b,d). Within‐plot variation in light penetration was not significantly related to the total and flowering abundances or richness of small species using either definition (Table S1).

**FIGURE 5:**
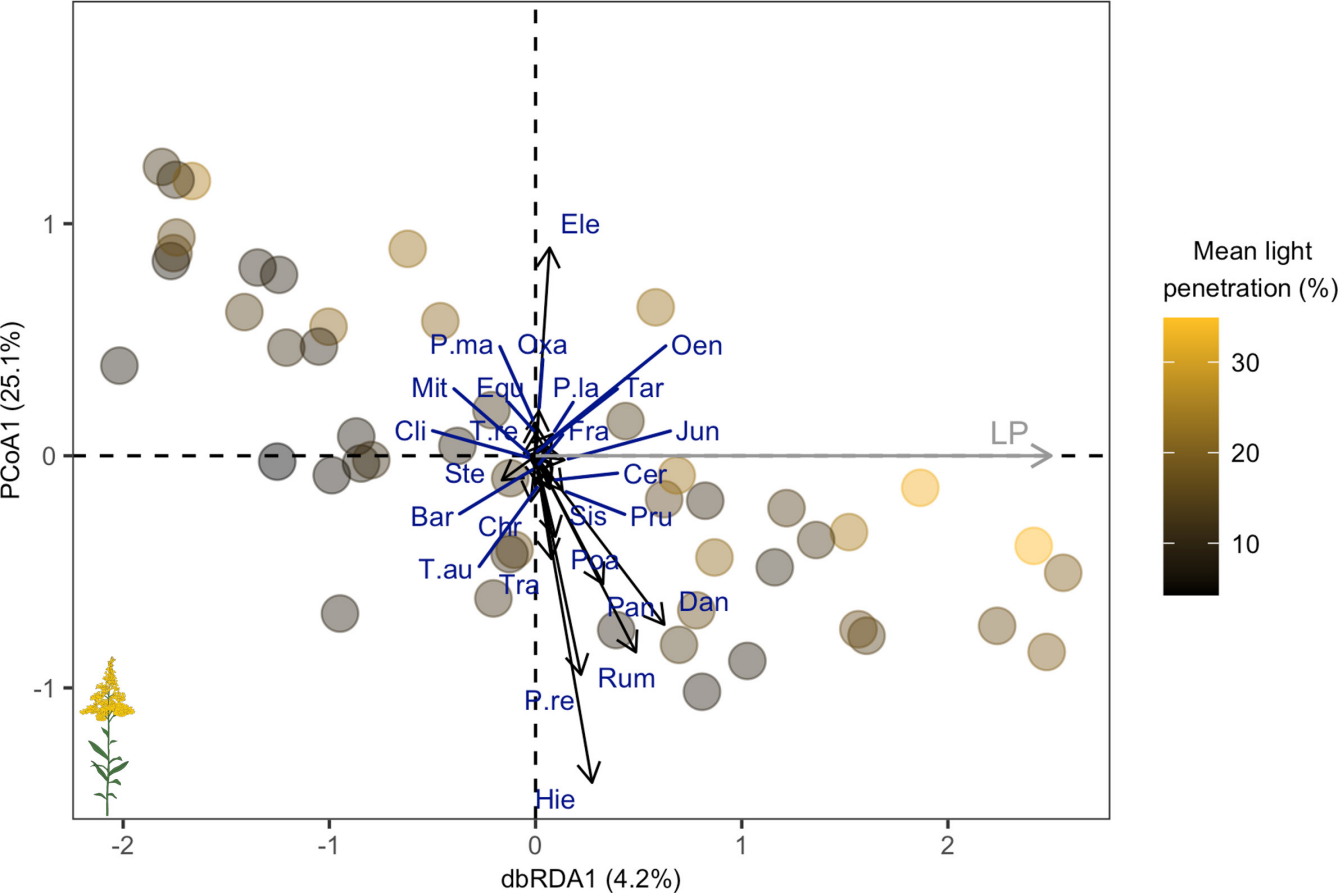
Distance‐based redundancy analysis (dbRDA) ordination for composition of flowering species (as indicated by the flowering icon) smaller than the median height (“median,” *N* = 26, <83 cm) with mean light penetration as a constrained axis. Light penetration (“LP,” gray arrow) explained 4.2% of the total variation in the flowering abundance of “median” species among plots (dbRDA1, *p* = .031, pseudo‐*F*_1,49_ = 2.06). Each point represents a plot. Points are shaded according to plot mean light penetration, ranging from black for the minimum mean light penetration, measured at 4.3%, to yellow for the maximum mean light penetration, measured at 34.9%. Species abbreviations are as follows: Bar *Barbarea vulgaris*; Cer *Cerastium fontanum*; Chr *Chrysanthemum leucanthemum*; Cli *Clinopodium vulgare*; Dan *Danthonia spicata*; Ele *Eleocharis compressa*; Equ *Equisetum arvense*; Fra *Fragaria virginiana*; Hie *Hieracium aurantiacum*; Jun *Juncus tenuis*; Mit *Mitchella repens*; Oen *Oenothera perennis*; Oxa *Oxalis stricta*; Pan *Panicum capillare*; P.la *Plantago lanceolata*; P.ma *Plantago major*; Poa *Poa pratensis*; Pot *Potentilla recta*; Pru *Prunella vulgaris*; Rum *Rumex acetosella*; Sis *Sisyrinchium montanum*; Ste *Stellaria graminea*; Tar *Taraxacum officinale*; Tra *Tragopogon pratensis*; T.au *Trifolium aureum*; T.re *Trifolium repens*

The composition of flowering small species defined using the “median” significantly changed with mean plot‐level light penetration (dbRDA1, *p* = .031, pseudo‐*F*_1,47_ = 2.06; Figure 5) but was unaffected by mean intraplot variation. Mean plot‐level light penetration explained 4.2% of the total variation in plot composition for flowering “median” small species. Eighteen species had higher flowering abundances associated with higher light penetration (as demonstrated by positive loadings along the light penetration axis), while eight species tended to have lower flowering abundances associated with higher light penetration (as demonstrated by negative loadings along the light penetration axis; Figure 5). Conversely, the flowering abundance and richness of “1st quartile” small species were unaffected by mean light penetration or mean intraplot variation; thus, neither was retained as axes.

## 4 DISCUSSION

### 4.1 Understanding variation in the light environment

To understand the effects of light limitation on small plant species in a herbaceous community, we first characterized variation in light penetration within plots (intraplot), among plots (interplot), and across 3 months of the growing season, when canopy shade was highest (temporal). Despite existing assumptions that light is not heterogeneously distributed within herbaceous communities, light penetration across plots in our community ranged from 0.3 to 72.4% (Figure S2A). Light penetration also varied within plots and across the 3 months we measured, and the degree of variance in light penetration among intraplot, interplot, and temporal measures was similar (Figure S3). Light penetration significantly decreased from June to July as well as from June to August but did not differ between July and August; thus, maximum canopy development was reached by July.

Analyses of light penetration through the canopy of natural herbaceous plant communities are rare (e.g., Kelly & Canham, 1992); those that exist are often conducted on communities that have been subjected to an experimental treatment. These include various fertilization (e.g., Borer et al., 2014; Semchenko et al., 2012; Urbas & Zobel, 2000), watering (e.g., Semchenko et al., 2012), mowing (e.g., Bachmann et al., 2018; Urbas & Zobel, 2000), herbivore exclusion (e.g., Borer et al., 2014; Izaguirre et al., 2006), and shading or illumination treatments (e.g., Izaguirre et al., 2006; Semchenko et al., 2012; Urbas & Zobel, 2000). Varied experimental treatments, along with differences among studies in how the light environment is quantified (e.g., 5cm from the ground by Kelly & Canham, 1992; 3–150 cm from the ground by Bachmann et al., 2018), make it difficult to compare data across studies and to get a general sense of light environments in herbaceous communities. However, when species richness was manipulated via seeding, analyses of old‐field and grassland communities have described ranges of percent light penetration similar to our community, with averages as low as 13% in 8‐species mixtures (Spehn et al., 2000), 20%–32% in 16‐species mixtures (Bachmann et al., 2018), and 3% in 32‐species mixtures (Spehn et al., 2000). Within our community, total species richness (i.e., the number of both flowering and nonflowering species of all heights) ranged from 13 to 29 among plots; thus, the ranges of light penetration and total species richness recorded in our non‐experimental study site are comparable to those in experimental study sites.

Because the herbaceous canopy lacks shrubs and trees, it is commonly speculated that light is both highly abundant and relatively homogenous within herbaceous communities. However, mean light penetration within our community (4.2%–35.9%) is comparable to light penetration in aspen, mixed wood, and old temperate forests (less than 25% at the ground level, Bartemucci et al., 2006). In some of our plots (Figure S2A), light penetration was also comparable to that experienced in tropical forests (less than 2% at the ground level, Valladares et al., 2002). Although herbaceous canopies are typically slower to close than forest canopies (Hallik et al., 2009), many small species in herbaceous communities persist throughout the season, when shade is highest (June–August). While shade tolerance is generally accepted as supporting the ability of small plant species to survive in forest understories, it remains an open question whether shade tolerance plays a similar role in herbaceous plant communities.

### 4.2 Testing hypotheses related to species size and light penetration

According to the size advantage hypothesis, larger species, in general, are competitively superior to smaller species, possibly due to their ability to intercept light while shading plants below them (e.g., Anten, 2005; Schwinning & Weiner, 1998; Tracey & Aarssen, 2014; Tracey et al., 2017). Consistent with this, we found that light penetration significantly decreased with mean plot height (Figure 3). Importantly, the height of species in sample plots, as measured here, left approximately 90% of variation in plot‐level light penetration unexplained. Some of this unexplained variation is inevitably due to the way in which we estimated plot height and the lack of a more graduated measurement of how light penetration diminished as it passed through the canopy. Future assessments based on measuring the heights of all plant species in plots, and additional light measurements at different heights within the canopy, would be of benefit, but were not possible for this study. Regardless, our results highlight that while tall species have the capacity to shade smaller plants, the biological significance of this advantage may be greatly reduced by the size distributions of tall species in plots, and by other factors that contribute to variation in composition within herbaceous plant communities. Inevitably, other factors, like available soil nutrients, can limit the height of the canopy by controlling which species can persist (Borer et al., 2014). Large species with large minimum reproductive size thresholds (Tracey & Aarssen, 2019) may be less likely to persist in low soil nutrient settings, and self‐thinning among tall plant species may create regular gaps, allowing light to reach smaller species (Schamp & Aarssen, 2014). Thus, nutrient limitation and competition among tall species may decrease the abundance of large plant individuals per area, simultaneously increasing light penetration and altering the importance of light as a previously limited resource. Herbivory can also impact canopy development directly as well as indirectly by influencing plant investments in herbivore defense.

We found evidence that the abundance and richness of small species are limited by light penetration; these patterns were consistent when we considered only flowering plants (i.e., when we excluded nonflowering plants from our analyses, as reported above) and when small species were defined as those below the median height of species in the community (i.e., “median” small species), but inconsistent when small species were defined as those in the first quartile of species heights in the community (i.e., “1st quartile” small species). These findings were less consistent across both definitions of small species (“median” and “1st quartile”) when we did not focus on flowering plants (i.e., when total abundance and richness data were used in our analyses, as reported in the Table S1). Importantly, the coefficients of determination (*R* ^2^) for regressions between light penetration and our response variables, when significant, ranged between 8% and 11% (Figure 4). Furthermore, only 4.2% of the variation in the composition of “median” flowering small species was explained by light penetration (Figure 5). This finding suggests that another factor, or combination of factors, could offer a better explanation of the variation in small species abundance in our community. For example, future work may consider the combined impact of light and moisture availability on small species distributions (Kelly & Canham, 1992; Knappová et al., 2013). Overall, low light penetration had a significant but meager impact on the flowering abundance and richness of small species in this community.

These results, in combination with the above finding that large species contribute only slightly to patterns of light penetration, provide useful context for understanding the conundrum that small species are numerous within crowded vegetation despite experimental evidence predicting that in such habitats they should be at a competitive disadvantage. First, light penetration to the ground level in our data was relatively low in July and August but was slightly higher in June. It is possible that some small species take advantage of higher light penetration prior to June (e.g., May), which would be consistent with observations that smaller plant species often flower earlier in the season (Du & Qi, 2010; Segrestin et al., 2020; Sun & Frelich, 2011). Second, within‐plot variation in light penetration had no significant impact on the flowering abundance and richness of small species at the plot level. This may be because plots with more variation in light penetration between subplots tended to be less shaded overall, potentially reducing the role of sunflecks as a critical source of light since shade was minimal (Figure S4). Third, the flowering richness of “1st quartile” species was no lower when light penetration was lowest, indicating that the flowering richness of small species under this definition was maintained under increasingly low light availability. The fact that the flowering richness of small species is only modestly impacted by light penetration suggests that many small species possess either shade avoidance or shade tolerance strategies which allow them to escape from or persist in shaded conditions, respectively.

Similar to these findings, evidence suggests that light penetration through forest canopies has a minimal impact on understory species richness (e.g., Bartemucci et al., 2006; Nicotra et al., 1999), abundance (e.g., Bauhus et al., 2001), and other measures of composition and diversity (e.g., Shannon‐Weaver diversity, evenness in Bartemucci et al., 2006). Forest‐based studies indicate that the abundance and richness of understory herb species are largely influenced by moisture (North et al., 2005) or dispersal limitation (Brudvig et al., 2011); the role of light is limited, with shade‐tolerant species being most impacted by drought (Kubiske et al., 1996). Shade‐tolerant species possess morphological, physiological, and biochemical traits that increase efficiency of carbon gain, allowing them to persist under low‐light conditions (Valladares & Niinemets, 2008). Shade tolerance is traditionally considered a survival strategy for various shrub and herb species under forest canopy shade (Canham et al., 1994) and is also associated with increased diversity of small species in the understory (Finegan, 1996; Toriola et al., 1998). Although understudied in herbaceous vegetation, shade tolerance may contribute to increased reproductive economy in smaller species (Aarssen, 2005, 2008). Adaptation to low light levels via phenotypic plasticity in shade‐tolerant traits may explain the persistence of small species richness under shade in our community (Ballaré, 1994; Hallik et al., 2009; Niinemets et al., 2015; Urbas & Zobel, 2000), as some individuals may express traits associated with more efficient light capture (e.g., high specific leaf area; Valladares & Niinemets, 2008), allowing less abundant species to persist. Future work should investigate the presence of shade‐tolerant traits under natural light conditions in herbaceous vegetation. Overall, the characteristics of the light environment in our community, and possibly the characteristics of many resident small species, ensure that any disadvantage they experience in competition for light is minimal, which may contribute to their persistence and richness within old‐field vegetation.

The effects of shading on the abundance and richness of small species were more consistently observed and stronger in general when we focused on flowering individuals (i.e., individuals which reached reproduction in the study year) in sample plots. This demonstrates that although “median” small species were able to persist under low light with no detected loss in total abundance or richness, some were not successful enough to reach reproductive maturity (i.e., a loss in flowering abundance and richness was detected). The presence of flowering individuals in a sample plot is evidence of a species’ capacity to successfully establish in that environment. Our results make it clear that light penetration does affect which small species can find success (i.e., regarding fitness), but its impact on the total abundance and richness of small species is relatively minor. Our results also support the contention that focusing censuses on flowering species can clarify plant community dynamics (Schamp et al., 2016; Schamp & Jensen, 2019).

## 5 CONCLUSION

The size of resident species in old‐field vegetation plots had a significant impact on light penetration; however, it appears that this impact is quite limited. This should be studied further, with more thorough assessments of plot‐level height and a more detailed assessment of light penetration to different levels of the canopy; this will clarify the degree to which large species have the capacity to shade smaller species in herbaceous communities. The flowering abundance and richness of small species were significantly lower when light penetration was lower; however, this impact was once again relatively small. Our results are consistent with the results of experiments that reveal a size advantage in competition, but add context by demonstrating that, in our study community, this advantage has only a very small impact on small species. Several possible explanations exist. For example, mitigating impacts, like herbivory, on large plants may reduce the capacity for large species to outcompete small species for light. It is also possible that one or more of several characteristics of small species (e.g., early season growth and reproduction, shade tolerance) contribute to greater reproductive economy (Aarssen, 2005, 2008). This study also adds to the sparse body of literature on the light environment in unmanipulated herbaceous plant communities.

## AUTHOR CONTRIBUTIONS

**Kelly C. Balfour:** Writing—original draft (equal); Writing—review & editing (equal). **Danielle A. Greco:** Formal analysis (lead); Writing—original draft (equal); Writing—review & editing (equal). **Riley Gridzak:** Writing—original draft (supporting); Writing—review & editing (equal). **Gillian Piggott:** Data curation (equal). **Brandon C. Schamp:** Conceptualization (equal); Methodology (equal); Writing—review & editing (equal). **Lonnie W. Aarssen:** Conceptualization (equal); Methodology (equal); Writing—review & editing (supporting).

## CONFLICT OF INTEREST

The authors declare that there is no conflict of interest.

## Supporting information

Table S1‐Fig S1‐S8

## Data Availability

Data used in these analyses are available on Dryad via: https://doi.org/10.5061/dryad.rn8pk0pcj.
