# Supplementary material for: Smaller species experience mild adversity under shading in an old‐field plant community
Source: Ecol Evol. 2022 Jun 22;12(6):e9006. doi: 10.1002/ece3.9006 (PMC9217891; doi:10.1002/ece3.9006)
Supplement: Supplementary file 1 — Table S1‐Fig S1‐S8 [file ECE3-12-e9006-s001.docx]

**Supplementary Material**

**Table S1.** Model summaries for mean plot-level species height and light penetration, and the abundance and richness of small species under two definitions. '1^st^ quartile' refers to all species smaller than the first quartile of species height for all species in the focal community (<53.25 cm). 'Median' species refers to all species smaller than the median height for all species in the focal community (<83 cm). The total census includes all rooted units within a plot (both flowering and nonflowering), whereas the flowering census includes only flowering rooted units within a plot. Light penetration refers to the mean light penetration per plot across June, July, and August. All analyses were run using linear models.

| Species group | Response | Census type | Transformation | Predictors | r^2^ | p |
| --- | --- | --- | --- | --- | --- | --- |
| — | Light penetration | Total | None | Mean plot height | 0.08 | 0.02 |
| 1^st^ quartile | Abundance | Total | Log_10_ | Light penetration | 0.09 | 0.02 |
| Median | Abundance | Total | Log_10_ | None | — | — |
| 1^st^ quartile | Richness | Total | Log_10_ | None | — | — |
| Median | Richness | Total | None | None | — | — |
| 1^st^ quartile | Abundance | Flowering | Log_10_ | Light penetration | 0.08 | 0.03 |
| Median | Abundance | Flowering | Log_10_ | Light penetration | 0.11 | 0.01 |
| 1^st^ quartile | Richness | Flowering | Log_10_ | None | — | — |
| Median | Richness | Flowering | None | Light penetration | 0.10 | 0.01 |


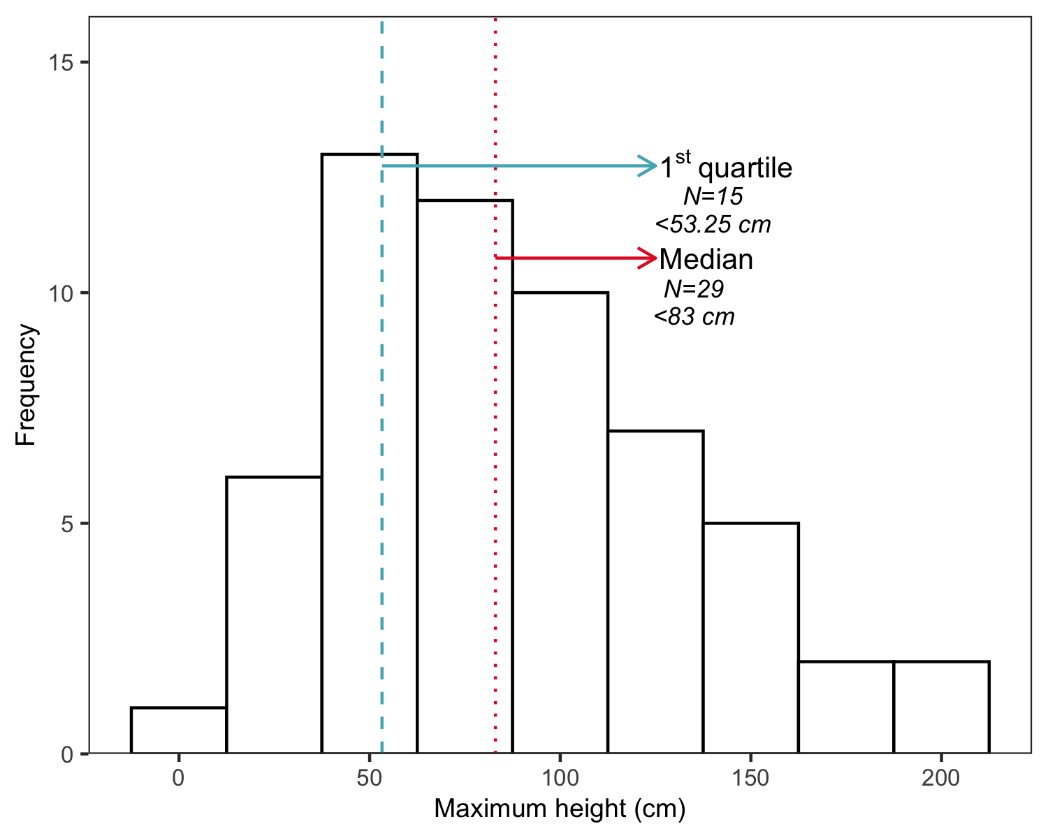


**Figure S1:** Frequency of maximum height (cm) values for species that occur in our old-field site (N=58). Vertical lines mark the cutoff points for each of the small species definitions: species below the first quartile (‘1^st^ quartile’; dashed light blue, N=15, <53.25 cm), and species below the median height (‘median’; dotted red, N=29, <83 cm).


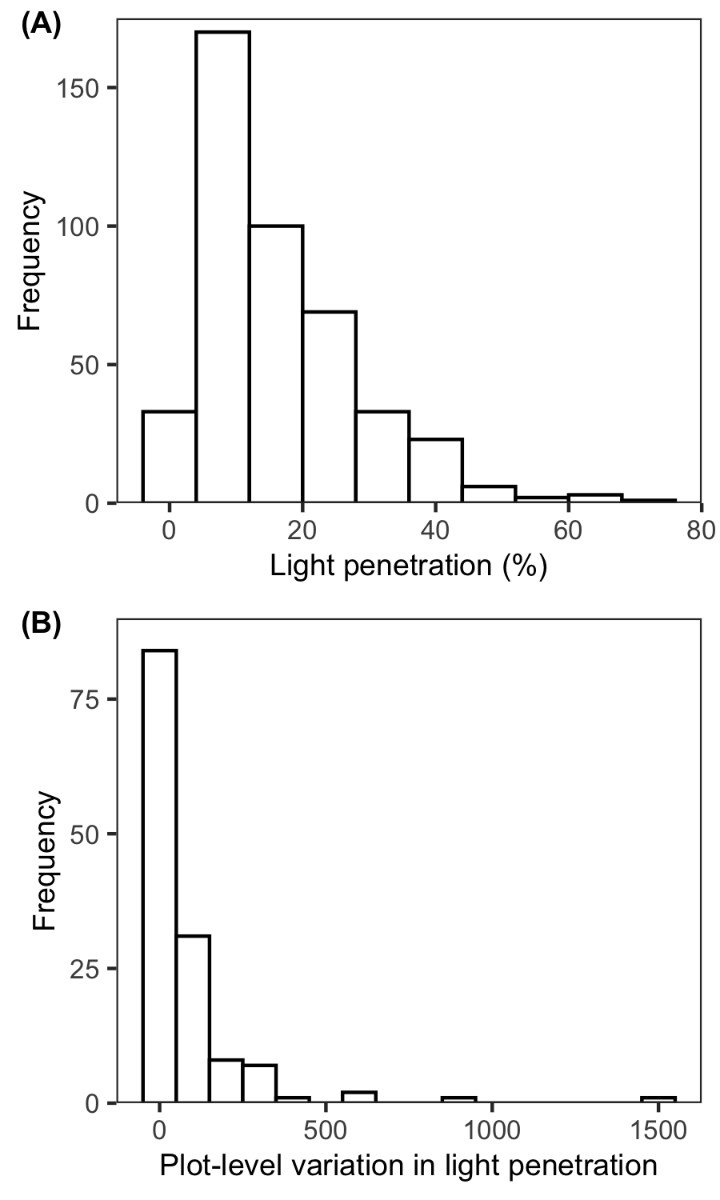


**Figure S2:** Frequency of light penetration (%; A) and plot-level variation in light penetration (B) values measured in our old-field site. Light penetration ranged from 0.29-72.4% (N=440, μ=16.6% ± 12; A) while plot-level variation in light penetration ranged from 0.03-1546.5 (N=147, μ= 82.8 ± 17.5; B).


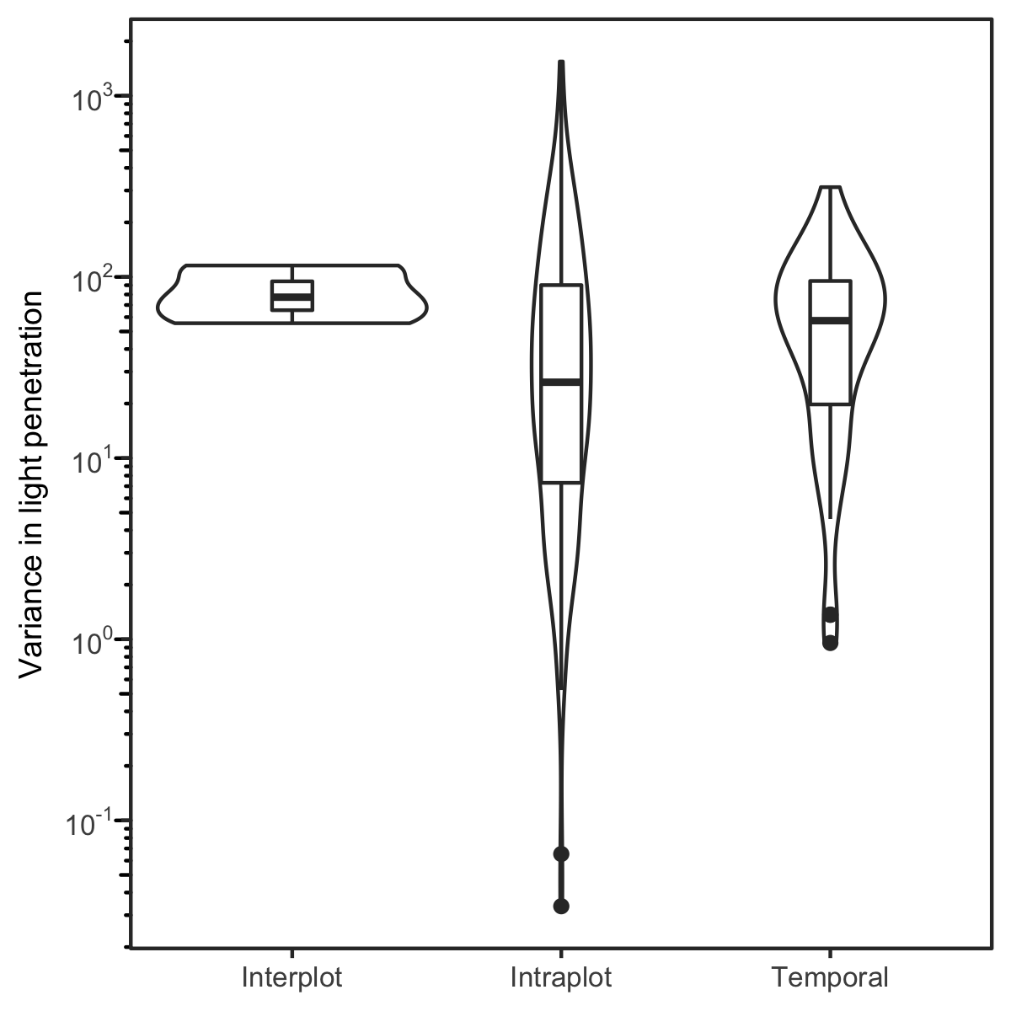


**Figure S3:** Interplot, intraplot, and temporally driven variance in light penetration (calculated as 100 × ground-level light intensity/above canopy light intensity; µmol s^-1^ n^-2^). For interplot variation, mean light penetration was calculated for each plot and the variance in these means was calculated for each month (N=3, median=77.3). To quantify intraplot variation, variance was calculated within each plot for each month (N=147, median=26.2). For temporal variation, mean light penetration was calculated for each plot and averaged across months (June, July, and August; N=49, median=57.4). Violin plots indicate the distribution of light penetration variance data. The y-axis was log_10_-transformed. Boxes represent the 25th to 75th percentiles of the data, and whiskers represent the 10th and 90th percentiles.


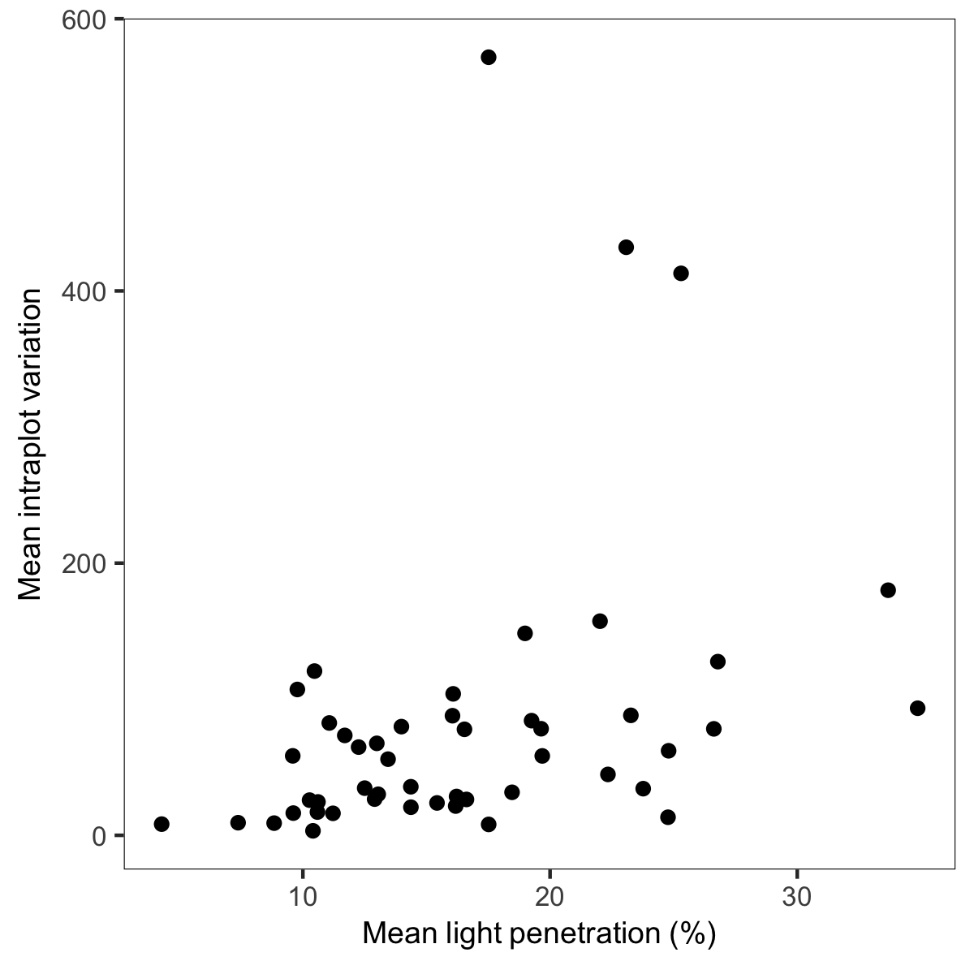


**Figure S4:** Mean intraplot variation in light penetration versus mean plot-level light penetration (%). Mean intraplot variation was calculated by determining the variance in light penetration within each plot for each month (June, July, and August) and taking the mean for each plot across months. Mean light penetration was significantly positively correlated with mean intraplot variation (Spearman correlation, p<0.001, rho=0.49).

**General Explanation of Total Census Results (Figures S5-S8)**

Total abundance measures (i.e., of both flowering and nonflowering rooted units) were less responsive to changes in light penetration. Only the total abundance of small species below the first quartile height (‘1^st^ quartile’) significantly increased with mean light penetration (p=0.021, r^2^=0.09; Fig. S6). Total abundance of small species below the median height (‘median’) and richness of both ‘1^st^ quartile’ and ‘median’ small species were not significantly affected by light penetration. Similar to the flowering census results (which included only flowering rooted units), mean intraplot variation did have a significant effect on the total abundance and richness of both ‘1^st^ quartile’ and ‘median’ small species.

In contrast, both ‘1^st^ quartile’ (dbRDA1, p=0.023, pseudo-F_1,47_=2.2; Fig. S7) and ‘median’ small species (dbRDA1, p=0.016, pseudo-F_1,47_=2.27; Fig. S8) community composition was significantly affected by mean light penetration. Mean light penetration explained 4.5% and 4.6% of the variation in the total abundance of ‘1^st^ quartile’ and ‘median’ small species, respectively. Eleven ‘1^st^ quartile’ species and nineteen ‘median’ species had positive loadings along the light penetration axis, reflecting an affinity for higher light plots, while four ‘1^st^ quartile’ and ten ‘median’ species had negative loadings along the light penetration axis, reflecting an affinity for lower light plots.

Finally, we examined the influence of light penetration on the incidence of individual species. The flowering abundances of each species was generally low, and no species were significantly affected by mean light penetration. However, total abundance of *Trifolium aureum* (30cm, p=0.002, r^2^=0.17; Fig. S9A), *Rumex acetosella* (40cm, p=0.009, r^2^=0.12; Fig. S9B), and *Danthonia spicata* (60cm, p<0.001, r^2^=0.2; Fig. S9C) significantly increased with mean light penetration.


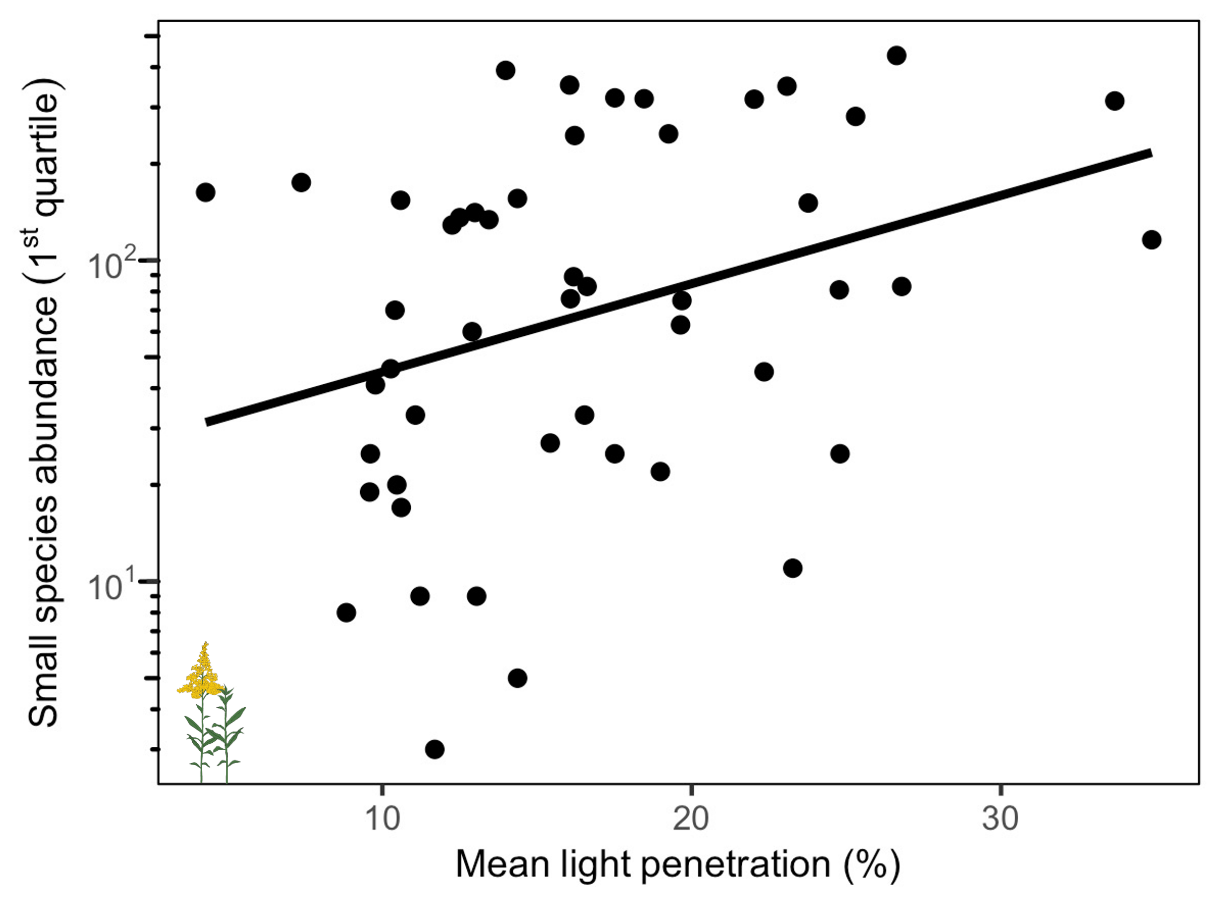


**Figure S5:** Total abundance (i.e., of both flowering and nonflowering rooted units, as indicated by the flowering and nonflowering icons) of small species under the first quartile height (‘1^st^ quartile’) versus mean light penetration (%) per plot. We used a log_10_ transformation on the total abundance of ‘1^st^ quartile’ small species. Total abundance of ‘1^st^ quartile’ small species significantly increased with mean light penetration (*P* = 0.021, R^2^ = 0.09).


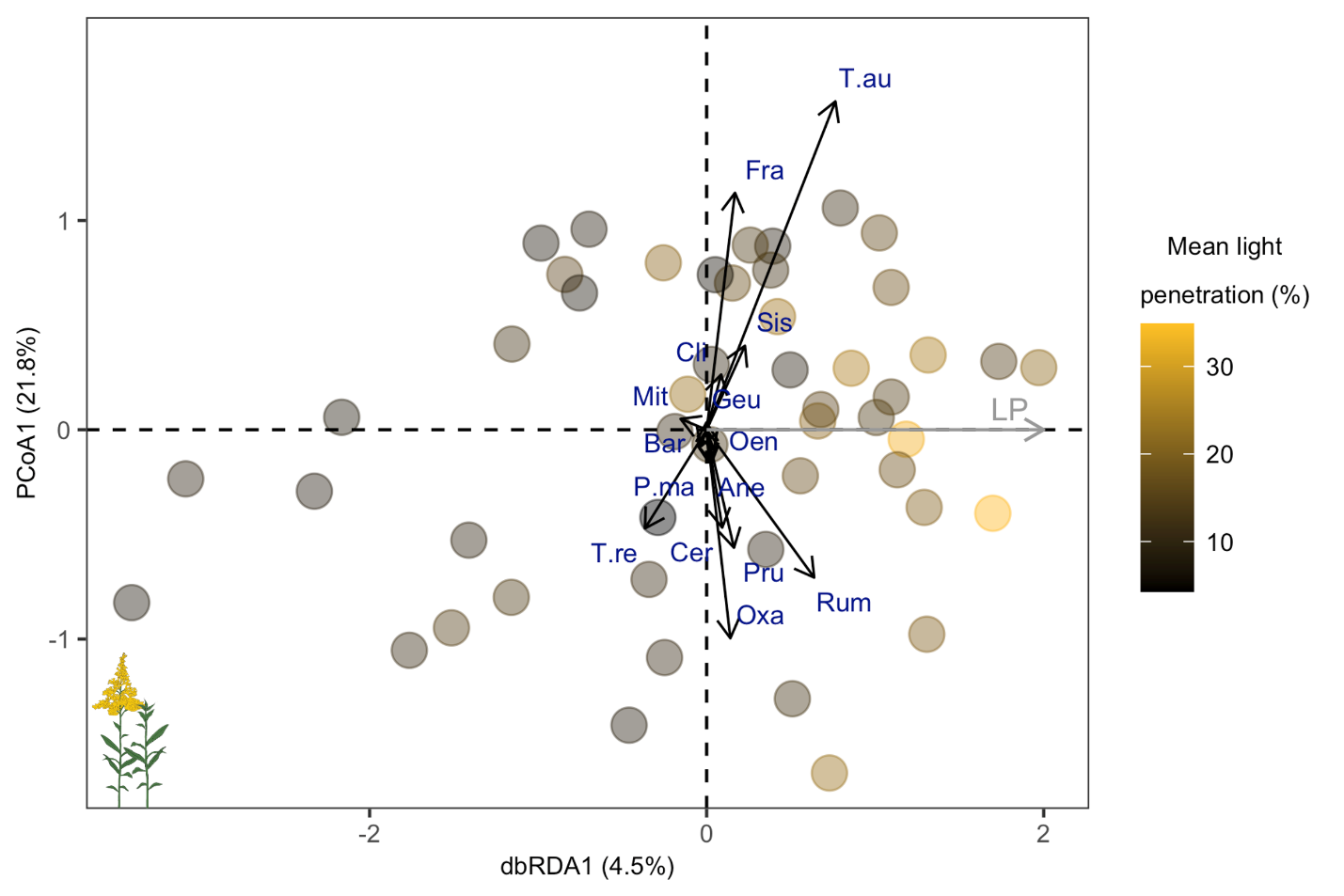


**Figure S6:** Distance-based redundancy analysis (dbRDA) ordination for composition of species smaller than the first quartile height (‘1^st^ quartile’; N=15, <53.25cm) with mean light penetration as a constrained axis. Light penetration (‘LP’, light grey arrow) explained 4.5% of the variation in the total abundance (as indicated by the flowering and nonflowering icons) of ‘1^st^ quartile’ species among plots (dbRDA1, p=0.023, pseudo-F_1,47_=2.2). Each point represents a plot. Points are shaded according to plot mean light penetration, ranging from black for the minimum mean light penetration, measured at 4.3%, to yellow for the maximum mean light penetration, measured at 34.9%. Species abbreviations are as follows: Ane *Anemone virginiana*; Bar *Barbarea vulgaris*; Cer *Cerastium fontanum*; Cli *Clinopodium vulgare*; Fra *Fragaria virginiana*; Geu *Geum aleppicum*; Mit *Mitchella repens*; Oen *Oenothera perennis*; Oxa *Oxalis stricta*; P.ma *Plantago major*; Pru *Prunella vulgaris*; Rum *Rumex acetosella*; Sis *Sisyrinchium montanum*; T.au *Trifolium aureum*; T.re *Trifolium repens*.


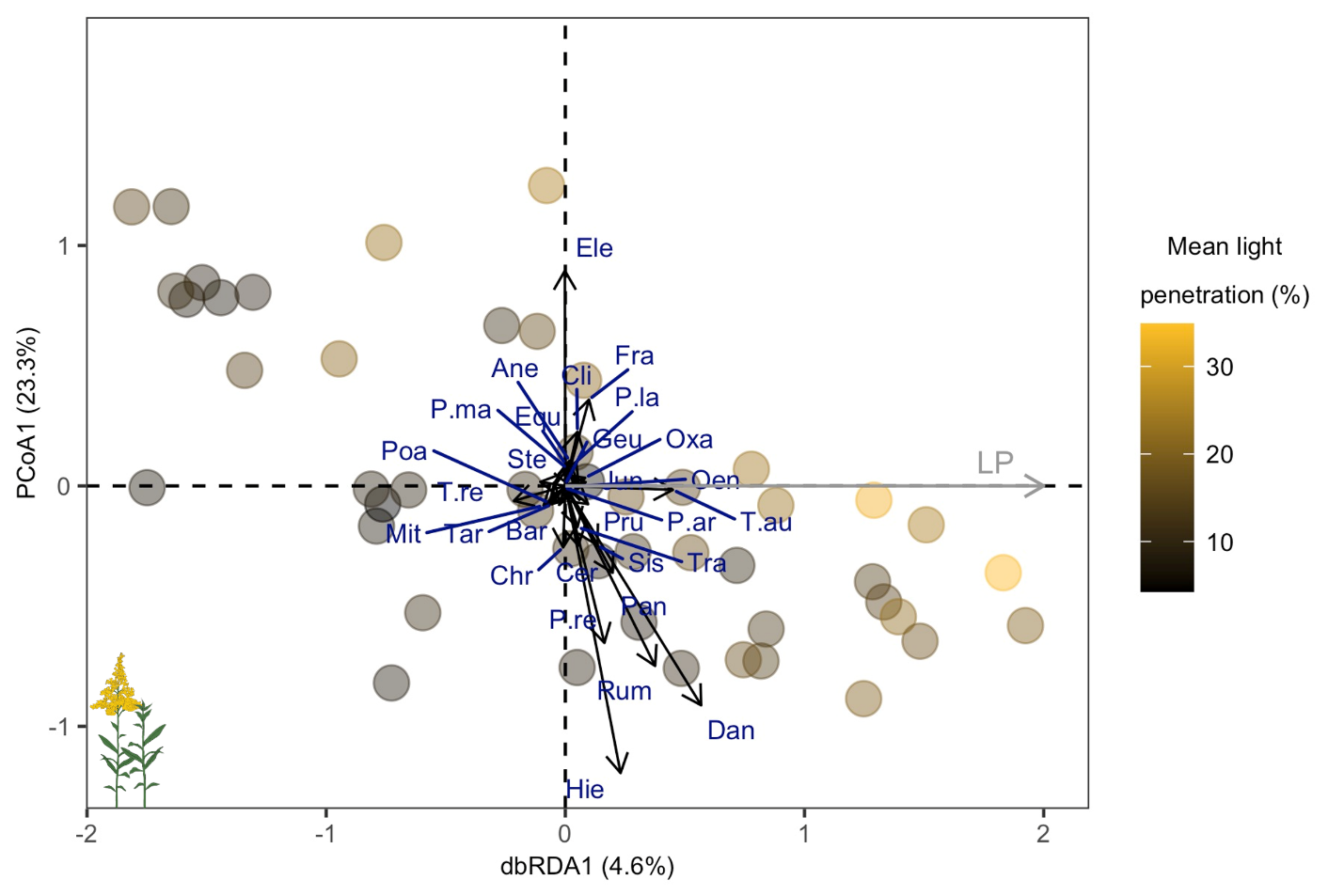


**Figure S7:** Distance-based redundancy analysis (dbRDA) ordination for composition of species smaller than the median height (‘median’; N=29, <83cm) with mean light penetration as a constrained axis. Light penetration (‘LP’, light grey arrow) explained 4.6% of the variation in the total abundance (as indicated by the flowering and nonflowering icons) of ‘median’ species among plots (dbRDA1, p=0.016, pseudo-F_1,49_=2.27). Each point represents a plot. Points are shaded according to plot mean light penetration, ranging from black for the minimum mean light penetration, measured at 4.3%, to yellow for the maximum mean light penetration, measured at 34.9%. Species abbreviations are as follows: Ane *Anemone virginiana*; Bar *Barbarea vulgaris*; Cer *Cerastium fontanum*; Chr *Chrysanthemum leucanthemum*; Cli *Clinopodium vulgare*; Dan *Danthonia spicata*; Ele *Eleocharis compressa*; Equ *Equisetum arvense*; Fra *Fragaria virginiana*; Geu *Geum aleppicum*; Hie *Hieracium aurantiacum*; Jun *Juncus tenuis*; Mit *Mitchella repens*; Oen *Oenothera perennis*; Oxa *Oxalis stricta*; Pan *Panicum capillare*; P.la *Plantago lanceolata*; P.ma *Plantago major*; Poa *Poa pratensis*; P.ar *Potentilla argentea*; P.re *Potentilla recta*; Pru *Prunella vulgaris*; Rum *Rumex acetosella*; Sis *Sisyrinchium montanum*; Ste *Stellaria graminea*; Tar *Taraxacum officinale*; Tra *Tragopogon pratensis*; T.au *Trifolium aureum*; T.re *Trifolium repens*.


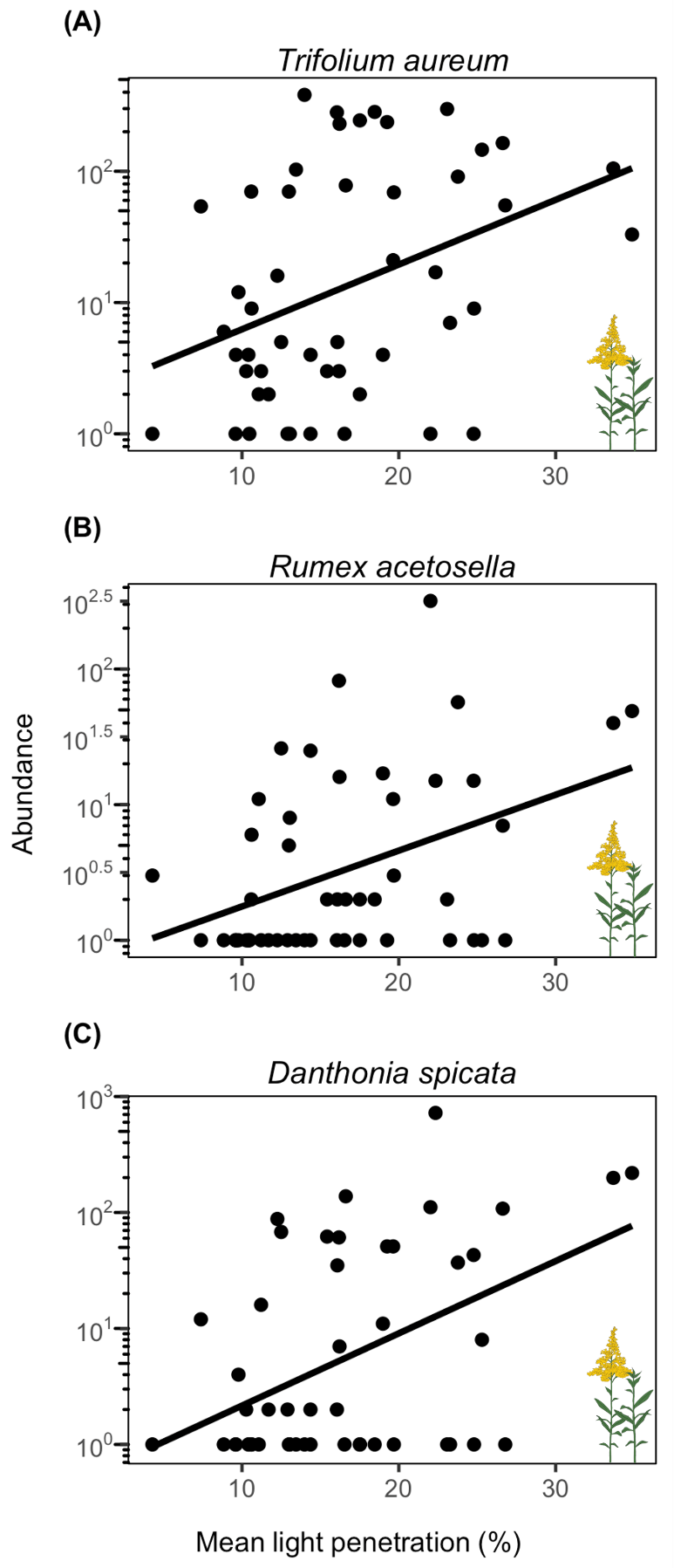


**Figure S8:** Total abundance (i.e., of both flowering and nonflowering rooted units, as indicated by the flowering and nonflowering icons) of *Trifolium aureum* (A), *Rumex acetosella* (B), and *Danthonia spicata* (C) versus mean light penetration (%) per plot. We used a log_10_ transformation on the total abundances of each of these species prior to modeling. *T. aureum* and *R. acetosella* are considered small by both definitions (‘1^st^ quartile’ and ‘median’), whereas *D. spicata* is only considered small using the ‘median’ definition. Total abundances of *T. aureum* (a, p=0.002, r^2^=0.17), *R. acetosella* (b, p=0.009, r^2^=0.12), and *D. spicata* (c, p<0.001, r^2^=0.2) significantly increased with mean light captured.
